# A very public replication of the temporal pattern to people's regrets

**DOI:** 10.1098/rsos.221574

**Published:** 2023-06-21

**Authors:** Jerry Richardson, Thomas Gilovich

**Affiliations:** Department of Psychology, Cornell University, Ithaca, NY 14850, USA

**Keywords:** replication, regret, judgement and decision-making

## Abstract

Most people recognize that mistaken actions generally sting more than equally mistaken and consequential failures to act (Gleicher *et al.* 1990 *Pers. Soc. Psychol. Bull.* **16**, 284–295 (doi:10.1177/0146167290162009); Kruger *et al.* 2005 *J. Pers. Soc. Psychol.* **88**, 725–735 (doi:10.1037/0022-3514.88.5.725); Landman 1987 *Pers. Soc. Psychol. Bull.* **13**, 524–536 (doi:10.1177/0146167287134009)). At the same time, most people have some intuitive appreciation of Whittier's claim that ‘For all sad words of tongue and pen, the saddest are these, “It might have been”’. As a result, few are surprised to learn that when people look back on their lives and identify what they regret most, they mention regrets of inaction significantly more often than regrets of action. Gilovich and Medvec (Gilovich & Medvec 1994 *J. Pers. Soc. Psychol.* **67**, 357–365 (doi:10.1037/0022-3514.67.3.357); Gilovich & Medvec 1995 *Psychol. Rev.* **102**, 379–395 (doi:10.1037/0033-295X.102.2.379)) identified the overarching pattern that incorporates both intuitions: regrets of recent vintage tend to centre on mistakes of action, but long-term regrets tend to involve failures to act. We conducted a replication of Gilovich and Medvec in the field using a unique source: a new museum in Chicago devoted to psychological science. We replicated the significant interaction between action/inaction and temporal perspective, but the precise pattern of that interaction diverged from that reported earlier.

## 1. Introduction

There are two common but competing intuitions about what people regret most in life. Most people recognize that mistaken actions generally sting more than equally mistaken and consequential failures to act. Suffering that results from changing jobs, switching sections of a class or altering a travel itinerary is generally more intense than when it results from sticking with the status quo [1–5]. At the same time, most people have some intuitive appreciation of Whittier's claim that ‘For all sad words of tongue and pen, the saddest are these, “It might have been”’. As a result, few are surprised to learn that when people are asked to look back on their lives and identify what they regret most, they mention regrets of inaction significantly more often than regrets of action [2,6–9].

Gilovich & Medvec [8,9] identified the overarching structure that incorporates both intuitions: recent regrets tend to centre on mistakes of action, but long-term regrets tend to involve failures to act. Gilovich and colleagues found support for this temporal pattern in people's reports of their biggest regrets in the near term and long term, in forced-choice assessments of what they regret most in the two time frames, and in the regrets implied by people's assessments of what they would do differently with the benefit of hindsight [1,2,8,9]. Their findings are echoed in studies that show greater regret over actions in the short term *or* greater regret over inaction in the long term [10–13] and in a recent report of a series of replication efforts involving four of the five paradigms used by Gilovich & Medvec [14].

Gilovich & Medvec [9] also provided evidence for the psychological processes responsible for this temporal pattern. People make more behavioural changes to deal with regrets of action than regrets of inaction, and they engage in more psychological work to deal with those regrettable actions that are not amenable to behavioural change. In addition, regrets of inaction do not diminish with the passage of time as much as regrets of action do because: (i) people often romanticize and exaggerate all the good things that would have happened on the road not taken and (ii) the worries and concerns they had that prevented them from acting at the critical time period seem less compelling with the passage of time, making their earlier inaction seem less justified.

Other studies of regret have examined the relative magnitude of regrets of action and inaction [15], the intensity of long- and short-term regrets operationalized as more or less than 1 year in the past [6], or they have altered the wording of the stimuli and dependent variable used by Gilovich & Medvec [9] to investigate how responsible respondents feel for their regrets [14].

To further explore the temporal pattern to regrets of action and inaction, we conducted a replication of Study 5 from Gilovich & Medvec [8]. Interestingly, Yeung & Feldman [14] replicated the temporal pattern reported by Gilovich and Medvec in three of the four paradigms they used in their series of replications, the one exception being Gilovich and Medvec's Study 5, making it an especially inviting target for the present replication effort. In that original study, adult participants from around town (Yeung and Feldman used MTurk respondents) were asked to think of (but not write down) their biggest regret of action and biggest regret of inaction from the past week, as well as the biggest regrets of both types over their entire lives, with the order of the time period counterbalanced across participants. Participants were then asked, for each time period, which they regretted more, the action or inaction regret. Gilovich and Medvec found that a significantly greater number of participants reported regretting their long-term inactions more than their long-term actions, but there was no significant difference in the number reporting one type of short-term regret or the other.

To test the reliability of this frequently cited result, we analysed data from an unusual (and, we believe, an unusually interesting) source. *Mindworks: The Science of Thinking* is an interactive science museum devoted to expanding the public's understanding of, well, thinking. Located right on ‘the Miracle Mile’ of Michigan Avenue in downtown Chicago, the museum has quickly become a popular destination for both local residents and tourists. Visitors can participate in a number of classic experiments in judgement, decision-making and psychological science more broadly. One exhibit invites participants to consider their biggest regrets.

## 2. Method

Anyone who participates in the regret exhibit is handed either a blue or red card. Those given a blue card are asked ‘When you look back on the last few days, what are your biggest regrets?’ They are then further asked to write down ‘something you DID that you now wish you had not done’ and ‘something you DID NOT do that you now wish you had done’. Those given a red card answer the same questions, but not about their biggest regrets of the last few days, but their biggest regrets in their ‘life to this point’. Both groups of visitors are then asked which they regret more, what they did or what they did not do, and to indicate their choice by placing their (blue or red) card on one of two pegs on the museum wall. Visitors can therefore see whether one colour, representing the short-term or long-term time frame (the independent variable), is more often placed on the regrettable action or regrettable inaction peg (the dependent measure). The cards are taken down each day, so visitors are not exposed to a substantial set of responses.

A description of this exhibit in the museum is posted on the *Mindworks* website, making it possible that some participants had visited the website and were aware of the purpose of the investigation beforehand. Note, however, that anyone who arrives at the *Mindworks* website has to click on at least two additional links to get to the description of the regret exhibit. The museum's director, furthermore, assured us that the ‘vast majority’ of museum visitors ‘come off the street’ and are ‘unfamiliar with behavioural science’. Although we cannot rule out the possibility that some volunteers' responses could have been influenced by the description of the exhibit posted on the website, considering the unplanned nature of most museum visits, the visitors’ unfamiliarity with behavioural science and the number of clicks necessary to find the hypotheses on the museum website, we believe it is unlikely.

Our replication adhered closely to the original study's stimuli, dependent variable (the forced-choice response) and data analysis. It differed from the original study in (i) sample size (to power the replication sufficiently); (ii) location (although both our study and the original involve US volunteers in the field); (iii) within-subjects versus between-subjects design (participants in the original study were asked about their regrets in both the short term and long term, whereas those in the replication were asked about one or the other); and (iv) the format of responses (participants in the original study responded orally whereas those in our replication wrote their responses on a card, which they hung—anonymously—on a wall). The within-subjects nature of the original study increased its power, but the vastly greater number of respondents in the replication (*n* = 2600 versus 32) more than compensated for that feature of the two designs. Methodological similarities and differences between [8], Study 5, and our replication are outlined in table 1.

**Table 1.** Differences and similarities between Gilovich & Medvec [8], Study 5 (the original study) and the replication study. Comparisons between the two studies are categorized as either different, close or exact.

comparison between original and replication studies			
study	[8]	current replication	comparison
sample size	*n* = 32	*n* = 2600	different
participants	locals in Ithaca, NY	anyone from anywhere in the world visiting the *Mindwise* museum in Chicago, IL	different
study location	in the field (bus stops and laundromats in Ithaca, NY)	in the field (visitors to *Mindwise* museum, Chicago, IL)	close
design	within-subjects	between-subjects	different
response format	oral	written	different
time period	looking back on life to this point (long term) versus the past week (short term)	looking back on life to this point (long term) versus the last few days (short term)	close
forced-choice response	more regret for either an action or a failure to act	more regret for either an action or a failure to act	exact
analysis	given the within-subjects design, a *z-*test on the difference in the number of participants who reported regretting their action more in the short term and their inaction more in the long term, and the number reporting the opposite pattern	given the between-subjects design, a *χ*^2^ test on the responses of all participants	close

The museum shared the responses of all the visitors who participated in this exhibit during its first year of operation (minus a trial period during the first few weeks when the cards were not collected or labelled as action or inaction)—2600 in all. Data collection began in mid-July 2021 and lasted until the end of July 2022. Eighty-four cards from the long-term condition and 77 cards from the short-term condition were not labelled as to whether they represented a greater regret of action or inaction and were therefore excluded from the data. The original study asked 32 respondents about both their biggest short-term and long-term regrets and found that, when considering their biggest short-term regrets, 53% (*n* = 17) said they most regretted an action they had taken and 47% (*n* = 15) said they most regretted an inaction. In the long term, in contrast, only 16% (*n* = 5) reported greater regret over an action taken and 84% (*n* = 27) reported greater regret over an inaction. We ran a *χ*^2^ sensitivity analysis for our replication based on our sample size (*n* = 2600 between-subjects participants) with a conservative estimate of assumed power (1 – *β* = 0.95) and found that we could reliably detect an effect size as small as *w* = 0.07 assuming *α* = 0.05. The code for this analysis can be found at https://osf.io/86nwp/?view_only=3d6c206971a94294b2a4445a1ec4dd97. A typical recommendation for replications is to use a sample size 2.5 times bigger than that used in the original study [16]. At over 80 times the sample size of the original, our replication is sensitive enough to detect quite small effects.

## 3. Results

A *χ*^2^ contingency test revealed a significant temporal pattern to respondents' reports of regrets of action and inaction in the long term and short term, *χ*^2^ (1, *n* = 2600) = 11.07, *p* < 0.001, *w* = 0.065, 95% CI [0.03, 0.10], replicating the main results reported by Gilovich & Medvec [8]. When asked to think about their biggest regrets over the past few days, significantly more participants (58%) (*n* = 743) said they were more troubled by their most regrettable action than their most regrettable inaction (42%) (*n* = 541), *z* = 5.67, *p* < 0.0001, 95% CI [0.55, 0.61]. But when asked to think about their biggest regrets in their ‘life to this point’, only 51% (*n* = 675) said they were more troubled by their most regrettable action, a percentage not significantly different from the null (*z* = 0.94, *p* = 0.35, 95% CI [0.49, 0.54]), whereas 49% (*n* = 641) indicated that they were more troubled by their most regrettable inaction.

This article received results-blind in-principle acceptance (IPA) at *Royal Society Open Science*. Following IPA, the accepted Stage 1 version of the manuscript, not including results and discussion, was registered on the OSF (https://osf.io/q9gfa/?view_only=205a60ca92d1489fbe2eb1d51a38a374). This registration was performed after data analysis. All conditions run and measures collected in the study are reported in this analysis. All data and code can be found at https://osf.io/86nwp/?view_only=3d6c206971a94294b2a4445a1ec4dd97.

## 4. Discussion and conclusion

Our study replicated the core finding reported by Gilovich & Medvec [8]—the extent to which people are troubled more by regrets of action or inaction varies predictably as a function of temporal perspective. In the present study, although the museum's visitors reported being more troubled by their biggest regrets of action in the short term, in the long term, there was no significant difference in the number reporting being more troubled by one type of regret or the other. Interestingly, the simple effects tests in the two studies yielded different patterns of significance underlying the reported interaction. Gilovich & Medvec's participants did not ‘favour’ one type of regret over the other in the short term, but a significant majority reported being more troubled by their biggest regret of inaction in the long term. By contrast, a significant majority of our participants were more troubled by their biggest regret of action in the short term, but as noted, there was no significant difference in the most troublesome type of regret in the long term. The present results, then, can be considered a successful replication in the sense that action regrets loom larger in people's minds in the short term than with the passage of time (or, alternatively, that inaction regrets loom larger with the passage of time). At the same time, the difference in the simple effects obtained by Gilovich and Medvec and in the present study means that the present effort should be viewed as a partial replication.

Why might the precise pattern of results differ across the two studies? It is impossible to know, but one obvious possibility is the inherent noise involved in conducting a study in a science museum, where the respondents read the instructions themselves rather than having them delivered to them by an experimenter, and where some did so by themselves and others in the company of who knows how many others. The greater noise involved in the present study may also be reflected in the smaller effect size of the observed interaction in the present study (*w* = 0.07) than in Gilovich and Medvec's study (*w* = 0.15).

Beyond that, what may have been responsible for the very substantial difference in the number of participants who said they were more troubled by their biggest regret of inaction in the long term in the present study (49%) and in the study reported by Gilovich and Medvec (84% of their 32 participants)? Here too we can only speculate, but one very plausible possibility centres on the likely age of most people who took part in the *Mindworks* exhibit.^1^ *Mindworks* is a family-friendly museum and parents want their children to learn from the visit and so they strive to get them to actively engage with the exhibits (Jamal, here's one I think you'll like!). The long term not being so long for younger respondents, there is less time for the processes that lead to greater regret over inaction to take hold. This leads to fewer lifelong reports of regrettable inactions among younger respondents (see [8, footnote 4]).

*Mindworks* is an outreach effort that strives to ‘give psychology away’ and inspire both critical thinking and greater knowledge of psychology on the part of the public. As the present results illustrate, the benefits of such public engagement efforts flow in both directions: They advance psychological science by enhancing the public's understanding of scientific psychology and they can provide useful data. In this case, the data support the central claim by Gilovich & Medvec [8] that whether people tend to regret mistakes of action or inaction depends on the time frame from which they are viewed.

## Data Availability

All data and R code used for analysis can be found on the Open Science Framework link here: https://osf.io/86nwp/?view_only=2cdadcc09abb49e49182a3df391c8b8a. This article received results-blind IPA at *Royal Society Open Science*. Following IPA, the accepted Stage 1 version of the manuscript, not including results and discussion, was registered on the OSF (https://osf.io/q9gfa/?view_only=205a60ca92d1489fbe2eb1d51a38a374). This registration was performed after data analysis. All conditions run and measures collected in the study are reported in this analysis. All data and code can be found here: https://osf.io/86nwp/?view_only=3d6c206971a94294b2a4445a1ec4dd97.

## Footnotes

1 Understandably, *Mindworks* did not have visitors who participated in this exhibit provide demographic information so we could not break down the results by age or any other demographic category.
